# A Physical Medicine and Rehabilitation Approach to Bilateral Hypoglossal Palsy After Orotracheal Intubation: A Case Report

**DOI:** 10.7759/cureus.28976

**Published:** 2022-09-09

**Authors:** Mafalda Oliveira, Ana Teixeira-Vaz, Antonieta Caldeira, Nilza Pinto

**Affiliations:** 1 Department of Physical Medicine and Rehabilitation, Centro Hospitalar Universitário de São João, Porto, PRT

**Keywords:** rehabilitation, dysarthria, dysphagia, swallowing, hypoglossal nerve palsy

## Abstract

Isolated hypoglossal nerve palsy is rare, usually unilateral, and typically associated with other neurologic lesions. Very few cases of bilateral hypoglossal nerve palsy have been reported. This report describes the case of a 34-year-old man who was admitted with community-acquired pneumonia and required invasive mechanical ventilation, after which severe tongue paresis, dysarthria, and dysphagia (Functional Oral Intake Scale (FOIS) 3) were reported. After the diagnostic workup, isolated cryptogenic bilateral hypoglossal nerve palsy was assumed, and a rehabilitation program was started. After hospital discharge, the patient presented with tongue atrophy; inability to elevate, protrude, or lateralize the tongue; dysarthria; and increased oral transit time with compensatory cervical extension when swallowing (FOIS 4). Four months after starting the rehabilitation program, there was evidence of improvement in tongue atrophy and mobility, along with a reduction of dysphagia severity (FOIS 6). About 10 months after starting the program, tongue mobility was almost normal, and the patient had a normal diet without limitations (FOIS 7). Despite the rarity of bilateral hypoglossal nerve palsy, this entity is associated with relevant functional impairments. A multidisciplinary approach to diagnosis and tailored rehabilitation programs are highly valuable in the management of these patients.

## Introduction

Isolated hypoglossal nerve palsy (IHNP) is a rare occurrence compared to other cranial nerve palsies, such as those involving the third, sixth, and seventh cranial nerves [1]. It is usually unilateral and generally associated with lesions of other cranial nerves or neurologic structures [2,3]. Bilateral hypoglossal involvement is an even more unusual finding, with few cases reported in the literature [4-7]. Many causes of IHNP have been reported in the literature, including malignancies, stroke, multiple sclerosis, sequelae of carotid endarterectomy, Guillain-Barré syndrome, head and neck trauma, post-central nervous system infection [8] post-neurosurgical and otolaryngologic procedures [9-12], bronchoscopy [13], use of a laryngeal mask airway, and subsequent to general anesthesia and orotracheal intubation [14-16]. In the former, nerve compression and overstretching could occur during position changes (including neck hyperextension for laryngoscopy and surgical positioning) [14-16].

This report describes a rare case of a patient with isolated bilateral hypoglossal paralysis (IBHP) after orotracheal intubation and the respective rehabilitation process.

This article was previously presented as a meeting abstract at the Virtual International Society of Physical Rehabilitation Medicine (ISPRM) 2021 Congress “Furthering Rehabilitation in a New World,” virtually held from June 12th to 15th, 2021.

## Case presentation

A 34-year-old male, working as a delivery man, with a history of hyper-immunoglobulin E (IgE) syndrome treated with omalizumab, was admitted to a community hospital due to community-acquired pneumonia. Due to insufficient response to antibiotics, with clinical and radiological deterioration, the patient was transferred to our tertiary care center six days later. The patient presented with severe bilateral pneumonia and pleural effusion, requiring ventilatory support three days after transfer, with invasive mechanical ventilation. The clinical response was favorable, and two days later, ventilatory weaning to non-invasive ventilation with a high-flow nasal cannula (60 L/minute) was implemented. Three days after ventilatory weaning, tongue paresis was reported (with absolute inability to actively mobilize the tongue to any quadrant). Dysphagia and dysarthria were also noted. A thorough diagnostic workup was then initiated. The laboratory analytic study (which included serologic studies to exclude infections, such as *Treponema pallidum* particle agglutination, human immunodeficiency virus, and *Borreliae*) yielded normal results, and the brain and neck magnetic resonance imaging (MRI) excluded parenchymal lesions. Assessment by an otolaryngologist showed no significant changes except tongue paresis. Flexible laryngoscopy assessment revealed discrete stasis in the piriform recesses, without any other changes in pharyngolaryngeal morphology or mobility, including at the level of the vocal cords. Electromyography was requested but it was not possible to perform it during hospitalization.

The patient was then evaluated by a Neurologist and a Physical and Rehabilitation Medicine (PM&R) doctor. By that time, the patient had an emaciated appearance, and, on neurological examination, there was evidence of severe dysarthria, with serious verbal articulation changes, characterized by distortion and articulatory inaccuracy of consonants in the production of phonemes /d/, /t/, /l/ and /r/, without dysphonia or other speech or language impairments. Moreover, there was a total inability to perform any tongue movements, without muscular atrophy or tongue fasciculations. Dysphagia for thin liquids and accumulation of spittle and food fragments in the oral cavity were also noted, with preserved ability to swallow pureed foods. Because the patient initially had a score of 3 on the Functional Oral Intake Scale (FOIS), a nasogastric tube for feeding was used for two days. Detailed neurologic examination revealed no evidence of other central, peripheral, or mixed nerve involvement. Therefore, IBHP with cryptogenic etiology was assumed.

A daily rehabilitation program was started with the collaboration of an experienced speech and language pathologist (SLP), which included exercises aimed at restoring tongue mobility, improving feeding functionality, and improving oral communication skills, as well as evaluation of the safety and efficacy of swallowing. The therapeutic intervention mainly included assessment and reeducation of orofacial motricity; isometric and isotonic myofunctional exercises, associated with cryotherapy, to stimulate the movements and strengthen tongue muscles; sensitive stimulation of the oral cavity; reeducation of verbal articulation, by identifying, teaching, and training the target phonemes, in an isolated position, in the context of words and in spontaneous speech; reeducation of tongue movements for swallowing; teaching of compensatory maneuvers for swallowing; and airway protective strategies.

After 21 days, the patient was discharged, and an outpatient PM&R appointment was scheduled. By that time, the patient showed improvement in the accumulation of saliva and the ability to swallow a pureed diet with thin liquids. Physical examination revealed some atrophy of the tongue, severe dysarthria, and an inability to elevate, protrude, or lateralize the tongue. In addition, there was the maintenance of an increased oral transit time associated with compensatory cervical extension when asked to swallow. No other cranial nerve deficits were found, and the remaining neurological examination remained unaltered. Given the remaining disabling deficits, a bi-weekly outpatient PM&R program with an SLP was initiated, aiming to continue the gains with the program initiated in the hospital. On the subsequent appointments, a slow but progressive improvement of tongue atrophy and mobility, mainly the protrusion, was found, despite maintaining low amplitude on lateral and elevation movements. Moreover, there was an improvement in speech articulation, maintaining difficulty with phonemes /r/ and /l/. Twenty-four weeks after starting the PM&R program, the patient still had mild dysphagia, scoring 6 on the FOIS, but was capable of oral feeding with a solid diet, with several adaptations such as excluding foods with potential adherence to the oral cavity walls, not ingesting hard and brittle foods or those with double consistency, as well as avoiding very dry food. Oral hydration with thin liquids was possible without any complications during feeding.

**Figure 1:**
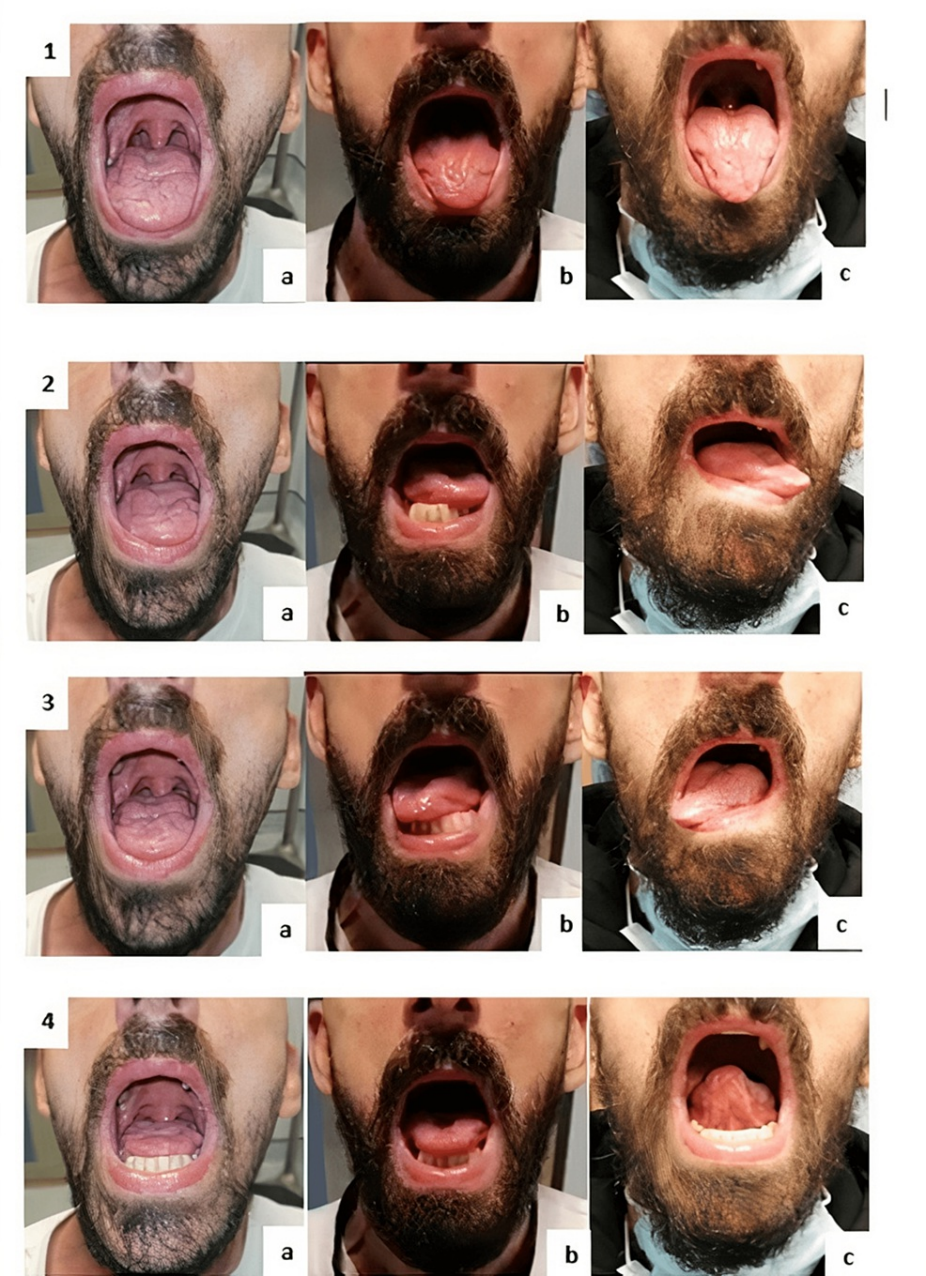
Evolution of tongue mobility during the rehabilitation program. Evolution of the different tongue movements with the rehabilitation program. Subfigure 1 represents the protrusion at the beginning (a) and after five (b) and ten (c) months of rehabilitation. Subfigure 2 represents left lateralization at the beginning (a) and after five (b) and ten (c) months of rehabilitation. Subfigure 3 represents right lateralization at the beginning (a) and after five (b) and ten (c) months of rehabilitation. Subfigure 4 represents the elevation of the tongue at the beginning (a) and after five (b) and ten (c) months of the rehabilitation program.

About 10 months after the event, the patient presented to the appointment with almost normal tongue mobility, doing a general diet without the need for food adaptation (FOIS 7), and normal verbal articulation (Figure 1).

On that date, the patient already presented the results of the electromyography, and the result was normal conduction at the level of the glossus muscles. There was no history of respiratory complications during the program. At this time, he was discharged from the treatments.

## Discussion

IBHP is a rare occurrence; hypoglossal palsies are usually accompanied by other cranial neuropathies [7]. While a number of etiologies have been proposed as causes of IBHP, none was found in this case, despite an extensive workup. In this case, the most plausible explanation was IBHP as a complication of the intubation/extubation procedure, possibly in relation to the neck position, compression by the orotracheal tube, or eventually pressure on the lateral roots of the tongue by the laryngoscope blade used in the intubation procedure [10,14]. Because the extracranial portion of the hypoglossal nerve is localized near a bone prominence in the transverse process of the first cervical vertebra, cervical hyperextension can cause nerve stretching and compression against the bone, with subsequent neuropraxia [13]. Another described mechanism relates to the inadvertent extubation of the trachea with the cuff inflated, leading to compression of the nerve against the greater horn of the hyoid bone [17]. The latter is also pointed out as a justification for the preponderance of male cases seen in the literature, given that the hyoid bone usually has greater absolute dimensions in male patients [5,18]. The presence of a calcified stylohyoid ligament, malformations of the skull base, and low blood pressure plus prolonged but unnoticed overinflation of an endotracheal cuff resting high in the larynx, just below the cords, are also reported as possible contributors to hypoglossal nerve compression or injury [7,19]. Indeed, most reported cases suggest the involvement of the extracranial section of the hypoglossal nerve [12,13].

Given the diagnostic challenge of this condition and the seriousness of its complications, early consultation with a multidisciplinary team is of paramount importance. An adequate approach to this kind of case, aiming at preventing any potentially life-threatening or long-term disabling conditions, requires the exclusion of central etiologies by a neurologist, the exclusion of oropharyngeal lesions by an otolaryngologist, and a formal dysphagia evaluation performed by PM&R doctors and an SLP. An early, tailored, and intensive PM&R program was performed, with a progressive clinical recovery, increasing the likelihood of this being a case of neuropraxia or axonotmesis of the nerve.

Usually, patients with this kind of lesion experience a good outcome, with the majority of cases having a self-limited course [5,7]. Nevertheless, this patient maintained symptoms until almost 10 months of follow-up, which is congruent with the data from several authors reporting that complete resolution may be achieved up to one year after the diagnosis [5]. However, it should be noted that publications about IHNP are still scarce.

In some cases, this entity can be severe enough to demand percutaneous endoscopic gastrostomy tube insertion for nutrition and to prevent aspiration [20]. Our patient could maintain oral feeding, despite the need for some adaptations. The electromyographic study was probably performed late, and we cannot assume what would be the result if it had been performed earlier; nevertheless, this could be a useful tool to strengthen the clinical suspicion of this diagnosis and guide prognosis, sometimes demonstrating pathological spontaneous activity of glossal muscles, consistent with an axonal lesion of hypoglossal nerves, or damage to the neural elements, not typical of transient neurapraxia [5,20].

## Conclusions

Even though IBHP is a rare finding, it should be considered in patients after transoral intubation with compatible complaints, such as dysphagia and dysarthria. This article reports a case of bilateral pathology of the twelfth pair, whose etiology could sometimes be a clinical challenge. Bilateral palsy could have a huge functional impact on patients. Adequate PM&R evaluation, tailored intervention, and follow-up are crucial.
